# Correlation between suicidal ideation and emotional memory in adolescents with depressive disorder

**DOI:** 10.1038/s41598-022-09459-4

**Published:** 2022-03-31

**Authors:** Shuwen Hu, Daming Mo, Pengfei Guo, Hongyu Zheng, Xiaolu Jiang, Hui Zhong

**Affiliations:** 1 Department of Child and Adolescents, Affiliated Psychological Hospital of Anhui Medical University, Hefei, Anhui China; 2 Anhui Mental Health Center, Hefei, Anhui China; 3 School of Mental Health and Psychological Sciences, Anhui Medical University, Hefei, Anhui China; 4 Department of Child and Adolescents, Hefei Fourth People’s Hospital, 316 Huangshan Road, Shushan District, Hefei, 230000 Anhui China

**Keywords:** Neuroscience, Psychology, Diseases

## Abstract

This study explored the differences in emotional memory between adolescents with and without suicidal ideation. Fifty adolescents with depression and suicidal ideation, 36 with depression but no suicidal ideation, and 41 healthy controls rated the emotional valence of positive, neutral, and negative pictures. Then, the recognition of the images was evaluated 72 h later. Adolescents with suicidal ideation reported more negative emotional valence scores for positive and neutral pictures and were significantly less likely to recognize negative pictures than were those without suicidal ideation. The performance of adolescents with suicidal ideation on the negative picture recognition test was closely related to anxiety, depression severity, and intensity of suicidal ideation. The negative bias toward neutral stimuli and cognitive impairment may be important risk factors for adolescents with suicidal ideation. Improving emotional memory via targeted management approaches may help young people with suicidal ideation.

## Introduction

Globally, suicide is the second leading cause of death among those aged 10–24 years anda leading public health problem^1^. A suicide generally includes suicidal thoughts, planning, attempt, and completion. Suicidalideation precedes suicidal behavior.Suicidal ideation is the most significant predictor of suicide attempt and completion. Longitudinal studies have shown that the greater the severity (high intent or planning) and pervasiveness (high frequency or duration) of the suicidal ideation, the higher the likelihood such ideation is to eventuate in an attempt. Suicidal thoughts,defined as thoughts of harming or killing oneself actively or passively, commonly occur in adolescents and more so among female adolescents^2^. In low- and middle-income countries, such as China, the main risk factors for suicide among adolescents include the female sex, bullying and violence, mental disorders, fragile family dynamics, and peer relationships^3^. Moreover, a longitudinal study of adolescents with aparental history of mood disorders found that depression was the strongest predictor of suicidal behavior in adolescents and young adults.The study also observed that children with depression were almost three times more likely to commit suicide before reaching puberty than late adolescents with depression^4^. Notably, female sex and suicidal ideation are predictive factors of suicidal behavior among adolescents^5^.

Adolescence (age of 10–19 years)—a unique formative period in which there are multiple physical, emotional, and social changes—is a critical period for the development and maintenance of social and emotional habits^6^. Promoting mental health and protection from adverse experiences and risk factors that may affect growth potential are essential aspects for physical and mental health in adolescence and adulthood. Suicide is a complex and multidimensional process that is influenced by many biological and environmental factors. Neuringer^7^ initially pointed out that cognitive ossification is a factor that significantly increases suicidal thoughts and attempts. Recently, a review^8^ of many studies showed that the impairment of neurocognitive functions was linked to the risk of suicide among adolescents and adults. In China, the incidence of suicidal ideation among adolescents with depression in 2021 was 38.2%^9^. Although suicidal ideation is a common precursor to attempted suicide, adolescents are more likely to "develop" actual behavior in the presence of impulsive aggression. The findings of a recent study indicate an association between higher errors of commission on the visual sustained attention task and increase in suicidal ideation in mood disorder patients before controlling for depressive symptoms^10^. Moreover, errors of commission on the attention test reflect impulsivity and appear to influence suicidal ideation by interacting with depressive symptoms.Attentional bias task automatically recorded commission errors and the reaction time to the stimulus. Accuracy rate was considered a measure of impulsivity or disinhibition when subjects reacted badly to non-targets^11^. Moreover, the concurrence of certain sources of acute stress, such as interpersonal conflict, may make adolescents more likely to act on their suicidal ideation^12^.

Negative emotional experiences may be more difficult to forget than neutral ones—a phenomenon known as the "emotional memory effect." Emotional memory represents the storage of information regarding survival experience. Neurons provide emotional meaning to environmental stimuli through an association mechanism. Both negative and positive emotional memories leave traces in the brain and strongly influence how we perceive the world and ultimately guide decision-making behavior. Emotional memory affects emotional intensity and emotional health. The relationship between emotional memory and suicide has been an interesting topic in the field of suicide research. Emotional memory^13^ (also known as emotional arousal event memory) is a type of explicit memory that involves the process of encoding, storing, retrieving, and extracting emotional information under certain circumstances. Emotional information includes the subjective experience of emotional, physiological, and behavioral responses to emotion and emotional stimulation. Studies have reported that some adults who attempt suicide have low thresholds for distraction and emotional sensitivity when dealing with environmental cues and that reduced memory function may also be associated with inappropriate organizational strategies or problems in obtaining information during the initial coding process, leading to increased vulnerability to suicide^14,15^. Moreover, in the Cries of Pain model of suicide^16^, frustration following a failed event leads to impaired emotional memory, compared with neutral stimuli, emotional stimuli take longer to process, and emotional stimuli will direct more attention resources to the processing links of memory tasks^17^, and bias in information processing is considered to be the basis for negative assessment of stressors and promotes negative memory schemas.This suggests the causal effect of specific negative emotional states on memory impairment. However, although most previous studies have focused on emotional memory, the impact of suicidal ideation on emotional memory is not yet fully understood.

Suicidal ideation and suicidal behavior are symptoms of adolescent depression, the prevalence of suicidal behaviour among Chinese adolescents increased before the age of 17 years^18^. Accumulating evidence indicates that neurocognitive deficits are an internal phenotype of suicide risk, and cognitive impairment hinders psychosocial function; however, most studies have focused on adults. Moreover, despite significant efforts made in recent years to prevent suicide among children and adolescents, the number of suicides is steadily increasing, and high levels of aggression and impulsivity are being highlighted as important characteristics of suicidal behavior among adolescents^19^. Notably, follow-up studies of attempted suicide by adolescents have revealed that they face a long-term and widespread risk of injury during adulthood, thereby creating or exacerbating social difficulties and problems in the future, regardless of the characteristics of their suicide attempt^20,21^. Memory may play an important role in the risk of suicidal behavior, possibly by preventing these individuals from using past experience to address current problems enabling them to looktowards the future^22–24^. Thus, it is necessary to examine the relationship between emotional memory and suicidal ideation at the early stage of childhood and adolescence to identify risk factors for suicidal behavior and design early interventions.

This study aimed to investigate the relationship between suicidal ideation and emotional memory in adolescents. Specifically, we combined the attentional bias task and emotional memory test to examine differences in attentional bias, emotional picture valence score, and recognition memory between adolescents with depression with and without suicidal ideation and healthy adolescents. We hypothesized that the group with suicidal ideation had positive emotional memory deficits (which were closely related to the intensity of suicidal ideation) compared with the group without suicidal ideation.

## Methods

### Participants

The sequential enrollment method was adopted to recruit patients with first-onset adolescent depressive disorders who were hospitalized in the Children and Adolescents Department of the Affiliated Psychological Hospital of Anhui Medical University between September 2018 and April 2021. The participants engaged in behavioral experiments and underwent psychological scale assessments. The inclusion criteria were meeting the diagnostic criteria for depression in the International Classification of Diseases, Tenth Revision (ICD-10), aged 13–18 years, and no intake of antidepressants or drugs known to affect cognition or cause withdrawal for over 2 weeks before enrollment. After excluding patients with hearing and vision problems, history of electroconvulsive shock therapy within 1 year, or history of suicide attempts, 86 patients with first-onset adolescent depressive disorder were included in the analysis. Based on the results of their evaluation using the Beck Scale for Suicide Ideation-Chinese Version (BSI-CV)^25^, the participants were divided into the following groups: participants with no suicidal ideation (n = 36) and those with suicidal ideation (n = 50).

Meanwhile, we also recruited age-, sex-, and education level-matched healthy young students from two ordinary middle schools in Hefei City, Anhui Province. The inclusion criteria were the answer “No” to items 4 and 5 of the BSI-CV (described below) and no neuropsychiatric disease and family history of suicidal behavior. The exclusion criteria were the same as those for the patient group. In total, 41 healthy adolescents were enrolled in the study.

### Informed consent and confidentiality

This study was reviewed and approved by the Ethics Committee of the Affiliated Psychological Hospital of Anhui Medical University (HSY-IRB-PJ-XJJ-ZH002), and All the participants and their guardians knew the content and purpose of the study, agreed and signed informed consent. I confirm that all methods are carried out in accordance with relevant guidelines and regulations.

### Measures

#### Psychological assessments

The BSI-CV consists of 19 items that assess the presence/intensity of suicidal ideation during the week prior to evaluation. Initially, five screening questions are administered for this assessment. If the answer to items 4 (active suicidal ideation) or 5 (passive suicidal ideation) is "weak" or "moderate to strong," then suicidal ideation is indicated; if the answer is "no," there is no indication of suicidal ideation. The internal consistency reliability and validity for measuring suicidal ideation of the BSI-CV are 0.944 and 0.368, respectively.

The Hamilton Depression Scale-17 (HAMD_17_)^26^ includes 17 items that assess the severity of depressed mood over the previous week. The Hamilton Anxiety Scale-14 (HAMA_14_)^27^ consists of 14 items that are used to evaluate the severity of anxiety over the previous week and has good reliability and validity for the evaluation of patients with depression.

The Mini-International Neuropsychiatric Interview (M.I.N.I.)^28^ is a psychiatrist-administered structured interview that assesses DSM-IV (Diagnostic and Statistical Manual of Mental Disorders, 5th edition) and ICD-10 psychiatric disorders. The M.I.N.I. B module is used to assess suicidality and current suicide risk and consists of 15 questions that are asked by an interviewer and answered with a yes or no. The first three questions assess recent suicide attempts in the previous month and the next 11 questions are about suicidal ideations, plans, or attempts in the past month. The final question asks if the patient has made any suicide attempts in his or her life. To assess the risk of future suicide attempts, we excluded the first three items from the score, and the rest were aggregated and classified as low risk (≤ 9 points), medium risk (9–16 points), or high risk (≥ 17 points).

According to the Columbia Classification Algorithm of Suicide Assessment^29^, a suicide attempt is defined as an attempt to end one's life with a certain degree of intent and as a self-injurious act that was committed without success and was confirmed to have occurred by a guardian and a competent physician, who then records a history of attempted suicide.

#### Attentional bias determination

We followed the dot-probe paradigm developed by Mathews and Tata^30^, referred to the test paradigm written by Professor Zhang Xuemin's team^31^, and used the E-Prime test software. Stimuli, including fixation points, of 16 face images and targets "E" or "F" were presented on a computer screen. The task was divided into four steps as follows: (1) a gaze point ("+") was presented for 500 ms in the center of a gray computer screen; (2) a face image (negative–neutral picture) simultaneously appeared on the left and right sides of "+" for 500 ms; (3) after the picture disappeared, the target "E" or "F" appeared on one side of the gaze point and the participants were required to place their left and right index fingers on the "E" and "F" keys respectively. During the test, the participants’ fingers were to be in contact with the keyboard. When the target appeared, the corresponding key was to be pressed as quickly and as accurately as possible for no more than 2000 ms; (4) a blank screen appeared for 1000 ms.

The measurement task included four exercises and 128 formal experiments, and it took approximately 6 min to complete. The facial stimuli used in the experiment were obtained from the Chinese Facial Emotion Picture System^32^. Half of the pictures (such as sadness and anger) were used as negative stimuli, and the other half (plain) were used as neutral stimuli for both male and female participants.

#### Emotional memory task

**Figure 1:**
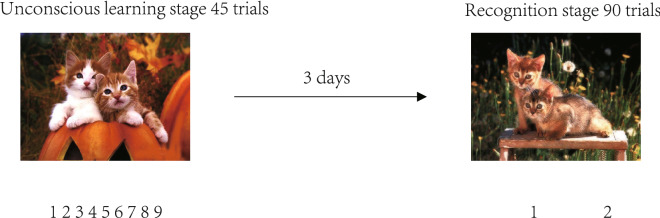
Emotional memory task procedure.

We designed an emotional memory task, referring to the method of Abrisqueta-Gomez^33^. In this study, the experimental stimuli included 90 emotional pictures divided into two groups. Each group included 15 positive valence pictures, 15 neutral valence pictures, and 15 negative valence pictures (Fig. 1). The first group of pictures was used as a memory coding task and the second group was used as interference pictures in the recognition test. In the unconscious learning stage, 45 pictures were randomly presented on the computer screen one by one, and each picture was presented for 3 s (the participants were not informed that content of the experiment should be memorized). The participants looked at the emotional pictures and were required to rate—on a scale of 1 to 9—the emotional valence of the pictures according to their current emotional state (with 1 representing the most negative state and 9, the most positive). There was no time limit to the interval for the participants to respond. In the recognition phase, after 72 h, the computer screen randomly presented two sets of mixed pictures. The task of each participant was to determine whether they were pictures he or she had seen three days prior, using "1" to indicate “yes,” and "2," “no.” The emotional picture stimuli used in the experiment were obtained from the Chinese Affective Picture System^34^.

### Statistical analysis

Chi-square test and analysis of variance (ANOVA) were used to compare demographic data, scale scores, picture valence scores, and recognition rates among the three groups. Kruskal–Wallis test was used to compare the number of misidentifications of pictures among the three groups, and multiple comparisons were then performed. Repeated-measures ANOVA was used to compare the response time and accuracy rate among different conditions to determine the main influence and interaction of the response time and facial stimuli. Pearson's correlation analysis was used to test the correlation between the recognition rate and the scale score in the patient group. A binary logistic regression analysis was performed to predict the risk of suicidal ideation. The level of statistical significance was set at p < 0.05. Bonferroni correction and corrected p-values were used to adjust the p-values while conducting the ANOVA.

## Results

### Psychological assessments

As shown in Table 1, the HAMD_17_ (p < 0.001) and HAMA_14_ (p < 0.001) scores of the suicidal ideation group were significantly higher than those of the no suicidal ideation and control groups. However, no significant differences in sex, education, or age were observed among the participants. The disease course and suicidal tendencies were the same in the groups with and without suicidal ideation.

**Table 1:** Key demographic and clinical factors in the (1) healthy group, (2) without suicidal ideation group and (3) with suicidal ideation group.

Characteristics	1 Healthy group N = 41 Mean (SD)	2 Without suicidal ideation group N = 36 Mean (SD)	3 With suicidal ideation group N = 50 Mean (SD)	t/χ^2^/F	p	Group differences
Male/female	17, 24	14, 22	12, 38	3.636	0.160	1 = 2 = 3
Education (year)	8.97 (1.50)	8.76 (1.50)	8.90 (1.30)	0.266	0.767	1 = 2 = 3
Age	14.83(1.44)	15.19 (1.59)	14.74 (1.53)	1.002	0.767	1 = 2 = 3
Course (month)	–	17.98 (3.20)	16.37 (1.51)	3.193	0.077	1 = 2 = 3
Suicidal tendency	–	25.53 (15.48)	28.56 (10.84)	− 1.676	0.099	1 = 2 = 3
HAMD_17_	0.00 (0.00)	14.78 (7.18)	20.98 (6.59)	160.763	< 0.001	3 > 2 > 1
HAMA_14_	0.00 (0.00)	12.28 (6.89)	19.20 (8.30)	103.657	< 0.001	3 > 2 > 1

–: no relevant data, HAMD_17_ Hamilton Depression Scale-17, HAMA_14_ Hamilton Anxiety Scale-1.

### Task performance

#### Attentional bias

The results of the attentional bias task are shown in Table 2. In this experiment, the control group responded faster to negative and neutral faces than did the groups with or without suicidal ideation. However, no significant differences in accuracy and attentional bias (neutral stimulus response time-negative stimulus response time) were observed among the three groups, and the main effect was not significant among different conditions [F(1,127) = 0.102, p > 0.05; F(1,127) = 3.536, p > 0.05].

**Table 2:** Response time and accuracy in attentional bias tasks.

Stimuli	Response time			F	p-values	Group differences	Accuracy rate			F	p-values	Group differences
	1 Healthy group	2 Without suicidal ideation group	3 With suicidal ideation group				1 Healthy group	2 Without suicidal ideation group	3 With suicidal ideation group			
	N = 41	N = 36	N = 50				N = 41	N = 36	N = 50			
Negative	606.24 ± 114.38	693.31 ± 125.75	712.13 ± 148.71	7.867	0.001	2 = 3 > 1	93.60 ± 4.66	93.97 ± 5.99	91.10 ± 9.40	1.937	0.148	1 = 2 = 3
Neutral	607.24 ± 118.21	687.38 ± 117.36	719.76 ± 155.32	8.185	< 0.001	2 = 3 > 1	93.29 ± 5.71	95.44 ± 5.56	92.03 ± 8.24	2.647	0.075	1 = 2 = 3

Response time: ms; accuracy rate: %

#### Emotional memory

**Figure 2:**
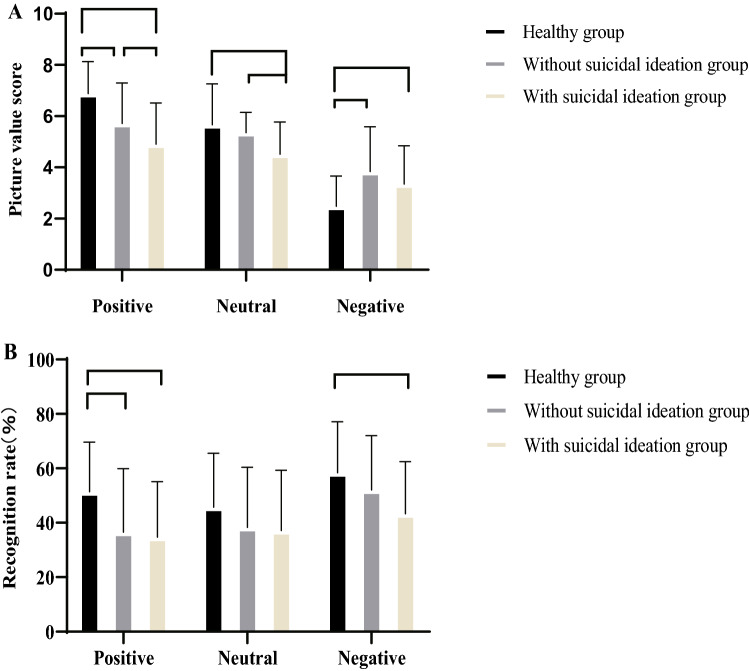
The valence scores and recogniton rates of positive, neutral, and negative emotion pictures were compared among groups. (**A**) Emotional picture valence score. (**B**) The accuracy of the picture recognition after 3 days.

Regarding the unconscious learning stage, when participants indicated pleasure elicited by the pictures with different valences, the positive score was lower in the patient groups than in the control group. The group with suicidal ideation had lower valence scores for the positive and neutral pictures than did the group without suicidal ideation. However, the negative picture valence scores were significantly higher in the group without suicidal ideation than in the control group (all p-values < 0.001) (Fig. 2A).

Regarding the recognition stage, the positive picture recognition rate (p < 0.01) was significantly lower in the patient groups than in the control group. No significant difference in the recognition rate of neutral pictures was noted among the three groups. The recognition rate of negative pictures in the group with suicidal ideation was significantly lower than that in the control group (p < 0.01), but no significant difference in the number of misrecognitions in each picture category was observed among the three groups (Fig. 2B).

### Correlations between the psychological scale score and recognition rate

In the emotional memory task test, the recognition accuracy of negative pictures was significantly negatively correlated with anxiety/somatization (r =  − 0.326), cognitive impairment (r =  − 0.309), mental anxiety (r =  − 0.302), and the intensity of suicidal ideation (r =  − 0.345) (Ps < 0.01), while the recognition rates of positive and neutral pictures were not significantly correlated with anxiety, depression, and suicidal ideation intensity (Table 3).

**Table 3:** Without suicidal ideation group and with suicidal ideation group correlation between accuracy of emotional memory test and scale scores (n = 86).

	Positive recognition rate	Neutral recognition rate	Negative recognition rate
**HAMD**_**17**_			
Anxiety/somatization	− 0.182	− 0.247	− 0.326*
Weight	− 0.152	− 0.181	0.014
Cognitive impairment	− 0.102	− 0.163	− 0.309*
Retardation	0.012	0.07	− 0.265
Sleep disorders	− 0.025	− 0.057	− 0.229
**HAMA**_**14**_			
Physical anxiety	0.005	0.026	− 0.273
Mental anxiety	− 0.015	0	− 0.302*
Suicidal ideation intensity	− 0.153	− 0.061	− 0.345*

*p < 0.01.

With suicidal ideation in adolescents with depression as a dependent variable (1 = Yes, 0 = No) and factors with statistical differences in single-factor binary logistic regression analysis (neutral score, mental anxiety, physical anxiety, cognitive impairment, and block) as independent variables, the binary logistic regression analysis model was run for risk factor analysis. The results showed that the neutral picture score of the emotional memory task was lower (p = 0.017, odds ratio [OR] 0.503, 95% confidence interval [CI] 0.286–0.883), while the cognitive impairment score of the HAMD_17_ factor was higher (p = 0.021, OR 1.388, 95% CI 1.051–1.833) as a predictor of suicidal ideation in adolescents with depression (Table 4).

**Table 4:** Without suicidal ideation group and with suicidal ideation group dual logistic regression analysis of risk factors for suicidal ideation (n = 86).

Risk factors	B	p-value	OR	95% SI	
				Low	High
**HAMD**_**17**_					
Cognitive Impairment	0.328	0.021	1.388	1.051	1.833
Retardation	0.1	0.571	1.106	0.781	1.565
**HAMA**_**14**_					
Physical anxiety	0.068	0.382	1.07	0.919	1.246
Mental anxiety	0.048	0.567	1.049	0.89	1.237
**Emotional** **memory**					
Neutral rating	− 0.688	0.017	0.503	0.286	0.883

Statistical significance was set at p < 0.05. *B* regression coefficient, *OR* odds ratio, *CI* confidence interval.

## Discussion

We found that regardless of the presence of suicidal ideation in adolescents with depression, their speed of response to the attentional bias tasks was slower than that of healthy adolescents. The patient groups had lower positive valence scores, lower recognition rates of positive pictures, and higher negative valence scores than did the control group. The group with suicidal ideation had a higher incidence of anxiety and depression than did the group without suicidal ideation. In the emotional memory test, the group with suicidal ideation had lower positive and neutral valence scores than did those without suicidal ideation, and negative picture recognition rates were lower in participants with suicidal ideation than in the control group. The accuracy of negative picture recognition was significantly negatively correlated with the total depression score, anxiety somatization, cognitive impairment, total anxiety score, degree of anxiety, and the intensity of suicidal ideation. Moreover, low neutral scores and high cognitive impairment scores were risk factors for suicidal ideation among adolescents.

Attentional bias can be parameterized into three dimensions as follows: individuals pay more attention to negative stimuli (attention to capture), selectively assign negative positions for long periods (attention to fixation), or assign attention to locations where negative stimuli do not exist (attention to avoidance)^35,36^. In the attentional bias task, adolescents with depression with or without suicidal ideation spent more time looking at negative faces and neutral faces; these findings are consistent with those in previous studies^37,38^. The findings suggest that lower performance in verbal memory and processing speed may be associated with a higher risk of suicide among adolescents, and they are more likely to focus on suicidal thoughts and suicide-related information in their environment. Although some studies^39^ showed similar differences in processing speed between patients with severe depression who had suicidal ideation and those who did not have suicidal ideation, these differences were not statistically significant; this may be explained by the association between lower scores of cognitive transfer and suicidal ideation^40^. However, our study did not observe significant differences in the attentional bias values among the three groups. This may reveal the relative specificity of neurocognitive processing in patients with different mental disorders; anxious participants exhibit a greater selective attention bias toward threat^41^, whereas no significant bias is observed in patients with depression. Previous studies have shown a significant correlation between suicidal ideation and daily memory coding (attention tracking), and daytime suicidal fantasies could explain the link between suicidal ideation and daily memory retrieval or impaired memory coding^42^. Moreover, a recent longitudinal study^43^ showed that those in early adolescence (ages 13–14) showed poorer accuracy in attentional tasks than did older adolescents (16–17 years old) and young adults (19–23 years old). Furthermore, Tavakoli et al.^44^ found a slight decrease in the accuracy of attention-target tests among adolescents with acute suicidal behavior than among heathy controls; Vivas et al.^43^ demonstrated that adolescence is a sensitive period during which attention and memory selection inhibition controls development, and the mechanism responsible for allowing one to effectively ignore distracting stimuli and to intentionally forget unwanted items is not fully established until the middle and late stages of adolescence. This might be due to the specificity of the selected population because the average age distribution of the three groups in this study was in the early adolescent years.

Depression and anxiety were more severe in adolescents with suicidal ideation than in those without suicidal ideation. Previous studies have consistently shown that adolescents with suicidal ideation have similar network structures to adolescents without suicidal ideation, and adolescents with suicidal ideation score higher on all depressive symptoms^45^; moreover, comorbidity of anxiety has shown to be protective against more carefully planned, high-fatality suicide attempts in patients with severe depression^46^; during a one-year follow-up^47^, people with suicidal attempts were 8 to 63 times more likely to present with cumulative comorbid disorders (e.g., mood disorders, abuse and dependence disorders, and anxiety disorder) than were people without experiences of psychopathology,although cognitive impairment was more pronounced under stressful and anxiety-provoking conditions, a feature that was identified as a significant contributor to suicidal thoughts and suicidal attempts^8^.

This study also found that depressed patients showed less pleasure in positive stimuli but increased pleasure in negative ones. Compared with those without suicidal ideation, those with suicidal ideation showed a stronger tendency to negatively evaluate positive stimuli. Negative bias in emotional processing is a major feature of depression, and it is evident even in high-risk people who do not have a history of personal depression, suggesting that it may be a key vulnerability marker for depression^48^. Similarly, the lack of state and idiosyncratic pleasure is only related to weakening of positive experiences and strengthening of negative experiences in patients with depression^49^. Patients with depression show flat emotional experience patterns of positive stimulation and have a stronger tendency to evaluate negative stimulation. In the general population, the diversity of negative and positive emotional experiences (emotional diversity) is associated with better mental health outcomes^50^. Chen et al.^51^ found that healthy participants were able to effectively reduce behaviorally and neurologically negative emotional responses through emotional autoregulation (which plays an important role in the neuropathology of suicide and self-mutilation) by re-evaluating negative emotional responses according to executive intent without increasing the involvement of cognitive control resources^52^. Moreover, those with suicidal ideation were more likely to interpret neutral pictures as negative pictures; these findings are consistent with those in the study by Maniglio et al.^53^ who found that the higher the depression component score in the emotional temperament dimension among healthy participants with a higher incidence of suicidal ideation and suicide plan, the higher the tendency for neutral facial expressions to be interpreted as sad facial expressions.

The emotional dimension theory identifies two dimensions of emotion, valence (positive–negative) and arousal (high–low), in which emotional arousal affects the memory^54^. We showed that the ability of adolescent patients to recognize neutral information unconsciously was similar to that of the healthy participants, although the patients tended to forget positive information. Meanwhile, the ability to recognize the negative content was significantly reduced in the group with suicidal ideation. Through the analysis of the correlation between the recognition rate of different valence contents and the intensity of anxiety, depression, and suicidal ideation, it was found that the recognition accuracy of negative pictures was related to the total score of depression, anxiety somatization, and cognitive impairment and that the intensity of suicidal ideation was significantly negatively correlated with the total score of anxiety; however, the recognition accuracy rates of positive and neutral pictures were not significantly correlated. Consistent with a previous study^55^, the healthy group showed more accurate memory for positive and negative emotional information, and memory responses were more assigned to negative pictures instead of neutral pictures. Only pictures with effective valence and high arousal were easier to remember than neutral information. Indeed, enhanced memory of emotional stimuli is an evolutionary adaptation that promotes human survival.

Cognitive neuroscience research with functional magnetic resonance indicates that the amygdala and hippocampus are interdependent in the encoding process of emotional memory in healthy people^56^. Activation of the amygdala is enhanced upon exposure to emotional stimuli. By recruiting stress hormones (i.e., norepinephrine)^57^ and corticosteroids to regulate the activities of the visual cortex, prefrontal cortex, and hippocampus, which affect explicit memory^58^, the amygdala’s regulatory effect on emotional arousal specifically acts on the hippocampus; the memory areas promote the consolidation process, leading to strengthening of memory traces, which in turn leads to more accurate memory performance of emotional content compared with neutral information. In addition, another resting-state functional magnetic resonance imaging study found that the amygdala–hippocampus/brain stem and amygdala–precuneus functions were impaired in adolescents with depression, and these circuits are crucial for different aspects of memory, self-processing, and emotions. These results suggest that memory processing may be impaired, causing the persistence of depressive symptoms in adolescents^59^. Abnormal activity of the amygdala is also related to suicidal ideation in adolescents^60^, and there is a stronger connection between the amygdala and emotional (happy, sad, or neutral) self-face recognition tasks in different suicidal ideation and suicidal attempt groups. During self-face processing, more rostral anterior cingulate cortex–left amygdala connections were associated with recent suicide attempts, while more rostral anterior cingulate cortex–right amygdala connections were associated with suicidal ideation. No significant difference in the number of misrecognitions in each category of pictures was observed among the three groups; the findings are inconsistent with those in previous studies. For instance, some studies have shown that patients with depression make more mistakes in recognizing emotion-sensitive words, especially words related to depression^61^, and suicide attempters make significantly more mistakes in the explicit recognition of facial expressions of disgust, although the errors are not related to the severity of depression and their ability to recognize certain social emotions decreased, which may impair the patients' ability to fully interact with their social environment, thus increasing the risk of suicide crisis. Some studies^62^ have also found that in the encoding process, the stress and high subjective arousal caused by negative events will simultaneously enhance emotional memory so that the most disgusting aspects of an event are well-remembered and then cause more resistance to misinformation, thereby decreasing the impact and number of misidentifications^63^. Among participants with a high state of anxiety, the number of awakenings decreases significantly^64^. When children and adolescents with anxiety disorders completed a spatial working memory and emotional stimulus-guided eye movement saccade task, no difference in working memory performance (reaction time and accuracy) was observed between the anxiety group and the control group, although the study did not simultaneously observe participants. Mueller^65^ has proposed two possible explanations for the degree of anxiety: (1) anxious children generalize attributes of fear faces as neutral faces, or (2) they simply shield the emotional valence and ignore these faces completely. The complexity of the relationship between anxiety and working memory depends on many factors, including the level of cognitive involvement and difficulty, the intensity of threat stimuli, and the individual's anxiety state. In general, the improvement of the short-term and long-term emotional states of adolescents is accompanied by an improvement in working memory ability^66^.

We found that the low scores for neutral pictures and high scores for cognitive impairment were risk factors for suicidal ideation in adolescents with depression. Picture valence has an interesting effect: When people view a picture, information from positive and neutral scenes accumulates in the memory at a constant rate, while information encoding from negative scenes is very slow at first but then gradually speeds up. Neutral scores are more easily interpreted as negative information than as positive information, and higher arousal leads to faster memory accumulation. Notably, memories of highly emotional events are malleable and easily distorted, and stress is regulated in different ways. The effects of reactivated emotional and neutral memory are long-lasting^67^, but patients with depression cannot inhibit neutral information from entering working memory and cannot inhibit and delete irrelevant information. Cognitive depression may be the basis of cognitive delay and attention deficit in patients with depression^68^. In the case of suicidal ideation, some people commit suicide, while others do not. Many external factors may play a role in the relationship between suicidal thoughts and suicidal behavior^69^, especially in emotional situations that promote such behavior. Previous studies^70,71^ have reported altered emotional memory biases in healthy volunteers from transcranial magnetic stimulation of the medial prefrontal cortex. These findings suggest that we should pay attention to the impact of emotional memory and cognition on the maintenance and development of suicidal ideation in adolescents with depression in clinical practice.

### Limitations

An important limitation constraining our interpretation of these data is that we only investigated healthy participants and patients with depression.Thus, it isunclear whether our results apply to populations with other mental disorders. Furthermore, this study's clinical cohort included hospitalized patients. In the case of patients without suicidal ideation, previous suicide attempts were not considered. Future studies with larger sample sizes are needed to compare emotional memory of past suicide attempts using a group of patients without current suicidal ideation. Moreover, although this study controlled for sex, age, education level, disease duration, and attentional bias to determine the effect of suicidal ideation on emotional memory in adolescents, there might still be other important co-occurring risk factors, such as childhood abuse^72^ the severity of psychotic symptoms and other significant life events.

## Summary

In conclusion, the relationship between emotional memory deficit (negative tendency to evaluate positive and neutral stimuli and significantly reduced cognitive ability to evaluate negative content) and suicidal ideation may reflect the unique contribution of emotional memory deficit to suicidal ideation in adolescents with depression. Emotional memory deficit may be an important risk factor for suicidal ideation in adolescents with depression. Our study indicates that emotional state and suicidal ideation are associated with emotional memory ability. Given that adolescents may be reluctant to disclose information regarding suicidal thoughts or behaviors to strangers and suicidal ideation is common among them, the emotional memory task can be used to identify adolescents who are at risk of suicide and require appropriate intervention. Further studies on emotional memory at different stages of suicide are warranted to screen and identify early emotional memory deficits in adolescents to enable early prevention of suicide risk.
